# Glutathione S transferase theta1 and mu1 gene polymorphisms and phenotypic expression of asthma in Egyptian children: a case–control study

**DOI:** 10.1186/1824-7288-40-22

**Published:** 2014-02-21

**Authors:** Nihal El Rifai, Nadia Moustafa, Nelly Degheidy, Manal Wilson

**Affiliations:** 1 Department of Pediatrics, Faculty of Medicine, Cairo University, Giza, Egypt; 2 Department of Clinical Pathology, Faculty of Medicine, Cairo University, Giza, Egypt; 3 New University Children’s Hospital (Abu El Reish), 4 – Gamal Salem St. Doki, Cairo, Egypt

**Keywords:** Asthma, Children, Egyptian, Glutathione S-transferase, Polymorphism

## Abstract

**Background:**

Asthma is the result of a complex interaction between environmental factors and genetic variants that confer susceptibility. The glutathione S-transferases (GSTT1 and GSTM1) are phase II enzymes thought to protect the airways from oxidative stress. Few and contradictory data are available on the association between asthma development and GSTT1 and GSTM1 polymorphisms in different ethnic groups. The current study aimed to investigate whether these polymorphisms are associated with asthma development in the Egyptian population.

**Methods:**

The cross-sectional study was performed on 94 asthmatic children 6 -12 yrs and 90 matched healthy controls. Candidates were subjected to clinical evaluation and measurement of absolute blood eosinophilic count, total serum IgE, and GSTT1 and GSTM1 genotype by multiplex PCR technique.

**Results:**

The results for GSTT1 null genotype were 87.2% and 97.2% for asthmatic children and controls respectively and showed to be significantly more in controls (P =0.007, OR:0.683, CI: 0.034 -0.715). The results for GSTM1 null genotype were 50% and 61.1% for asthmatic children and controls respectively and showed to be nonsignificant (p = 0.130, OR: 1.000, CI: 0.54- 1.86). Also, no association was detected between GSTT1 and GSTM1 polymorphisms and atopic conditions or asthma severity.

**Conclusion:**

The significant detection **o**f GSTT1 null genotype more in controls than in asthmatics with no association with other atopic manifestations or asthma severity and the lack of association detected between GSTM1 polymorphism in relation to asthma, atopy or asthma severity confirm the uncertain role of those genes in the development of asthma.

## Introduction

Asthma is a disorder of the airways characterized by several symptoms such as airflow obstruction, airway inflammation, and hyper responsiveness [1]. The study of genetic factors involved in complex pathologies such as asthma is arduous, not only because of human genetic variability, or incomplete penetrance, but also because, in complex disease studies, the importance and strength of gene to gene and gene to environment interactions need to be considered [2]. The prevalence of candidate gene polymorphisms for asthma varies considerably worldwide, and accordingly, ethnicity should be considered as a factor that might act on and influence asthma development. Previous data based on intra- and inter-population frequency differences suggest that the association between a given genetic polymorphism and asthma cannot be extrapolated from one ethnic group to another [3].

Phase II detoxification enzymes, particularly classes of GSTs, play an important role in inflammatory responses triggered by xenobiotic or reactive oxygen compounds [4]. The GSTM1 and GSTT1 are two important phase II enzymes that protect the airways from oxidative stress [5]. They utilize as substrates a wide variety of products of oxidative stress [6]. The inability of GST variant enzymes to detoxify reactive oxygen species contributes to the activation of the inflammatory process, bronchoconstriction, and the exacerbation of asthma symptoms [4]. In particular, GSTM1 and GSTT1 null polymorphisms may influence the pathogenesis of respiratory diseases. Numerous studies have documented associations between genes implicated in the oxidative stress response and respiratory phenotypes, but data suggest that they may not be consistent across ethnic groups owing to differences in intra- and inter-ethnic allele frequencies [7].

The aim of the current study was to detect the presence of an association between GSTM1 and GSTT1 polymorphisms and asthma, atopy or asthma severity.

## Methods

The present cross-sectional case–control study is conducted on a group of Egyptian asthmatic children (n: 94) and their age and sex matched healthy controls (n: 90) from September 2012 to June 2013. Patients were recruited from the allergy clinic of Cairo university specialized pediatric hospital where they were following up after being diagnosed according to GINA guidelines criteria of asthma classification [8]. All patients were subjected to a questionnaire containing a detailed history and clinical examination with emphasis on age, sex, family history, presence of atopic manifestations and asthma severity classification according to GINA Guidelines [8]. The following investigations were performed for all patients and controls.

### Total immunoglobulin E (IgE) and Prick test assays

Atopy was defined by a positive history of atopic manifestations, positive skin prick test (wheal diameter ≥3 mm) to at least one of the following aeroallergens (Dermatophagoides Farinae, hay Dust, Dermato-pteronyssinus, Alternaria Tenuis, Moulds II, Candida Albicans, Cat epithelia, Hen’s egg, Dog epithelia, Grasses/cereals, Cow’s milk in the presence of positive histamine control and negative physiological saline control using reagents obtained from Allergopharma D21462 Reinbek, Germany) and by the quantitative determination of human total IgE in serum using the DiaMed Eurogen IgE quantitative technique which is a monoclonal antibody based enzyme immunoassay (Positive values were taken to be ≥200 IU/ml). Among the 94 asthmatic children there were 67 atopic and 27 nonatopic children.

### Pulmonary function tests assay

Spirometric measurements using a Jaeger Master Screen Spirometry system (Jaeger Co) were done and included forced vital capacity (FVC), forced expiratory volume in 1 s (FEV1), and forced expiratory flow between 25% and 75% expired volume (FEF25-75). Short-acting bronchodilators were stopped at least 8 h before the test. All pulmonary function data were collected at a single visit. A minimum of 3 results within 10% of each other were recorded, and the result with the highest FEV1 was analyzed. The participants were not suffering from asthma exacerbations or other acute illnesses at the time of the measurement of pulmonary function. The lung function test results were expressed as a percentage of that predicted.

The personal, family, medical history and clinical presentation of controls were free of any atopic or allergic diseases with negative skin prick tests, normal total IgE values, normal lung function tests.

### Genotyping of GSTT1 and GSTM1

100 ng of DNA were amplified in a-50 ul multiplex reaction mixture containing 0.90 pmol of each of the following GSTT1 primers (GSTT1-Forward: GAACTCCCTGAAAAGCTAAAGC and GSTT1-Reverse: GTTGGGCTCAAATATACGGTGG) and GSTM1 primers (GSTM1-Forward: TTCCTCACTGGTCCTCACATCTC and GSTM1-Reverse: TCACCGGATCATGGCCAGCA). As an internal control, the beta-globulin gene was also amplified using the following amplification sequence (Forward primer: GCCCTCTGCTAACAAGTCCTAC and Reverse primer: GCCCTAAAAAGAAAATCGCCAATC) [9]. The amplification reaction consisted of 0.9 pmol of each primer added to 12.5 u PCR master mix which contains 3.5 mM MgCl2, 200 uM dNTPs, 5 ul 10X PCR buffer, and 2U TaqDNA polymerase (Fermentas).

The PCR protocol included: initial melting temperature of 94°C (5 minutes), amplification by 35 cycles of 20 seconds at 94°C, 20 seconds at 64°C, and 30 second at 72°C) then final extension at 72°C for 7 minutes. Analysis of PCR products on agarose gels where a fragment of 215 pb indicated the presence of GSTM1, a fragment of 480 pb indicated the presence of GSTT1 and a fragment of 280 pb indicated the positive internal control B globulin. The subjects were classified as either (+), when at least one specimen of the gene was detected, or (-) when they showed a null genotype.

### Ethical considerations

The aim and nature of the study was explained for each candidate and/or parent before inclusion. An informed written consent was obtained from parents/surrogates before enrollment. Children old enough were asked for consent. Cairo University Hospital Research Ethical Committee approved the work and it conforms to the provisions of the Declaration of Helsinki in 1995 (as revised in Seoul 2008).

### Statistical analysis

Data were statistically described in terms of mean ± standard deviation (± SD), median and range, or frequencies (number of cases) and percentages when appropriate. Comparison of numerical variables between the study groups was done using Student’s *t* test for independent samples when comparing 2 groups of normally distributed data and Mann Whitney test when comparing 2 groups of non-normal data. Kruskal Wallis test with posthoc multiple 2-group comparisons was used to compare numerical data between more than 2 groups. For comparing categorical data, Chi square (χ^2^) test was performed. Exact test was used instead when the expected frequency is less than 5. Odds ratio with its 95% CI was used to present the relation between haplotypes in cases and controls. Haldane modification was used when the occurrence of any haplotypes was zero. *p* values less than 0.05 was considered statistically significant. All statistical calculations were done using computer programs SPSS (Statistical Package for the Social Science; SPSS Inc., Chicago, IL, USA) version 15 for Microsoft Windows.

## Results

We investigated 94 children with asthma, approximately 67% of whom were atopic, and 90 age and sex matched healthy controls. Table 1 summarizes the characteristics of both groups. Age was found to be significantly higher in controls (p = 0.025). Male sex was found to be significantly higher in asthmatics (p = 0.019). No significant differences were found between asthmatic patients and healthy controls for any of the other characteristics analyzed. Data regarding the genotype frequencies of the GSTT1 and GSTM1 homozygous deletions in asthmatics and controls are also shown in Table 1. GSTT1 null genotype was found to be significantly higher in controls than in asthmatics (p = 0.007, CI = 0.683, OR = 0.034-0.714). No significant difference in the genotype distribution of the GSTM1 gene was found between asthmatics and controls. No significant differences in the genotype distributions of the GSTT1 or GSTM1 genes were found between atopic and nonatopic asthmatics (p = 0.706 and 0.820 respectively) as shown in Table 2. Also, no significant differences in the genotype distributions of the GSTT1 or GSTM1 genes were found between asthmatics stratified by disease severity (p = 0.236 and 0.892 respectively) as shown in Table 3.

**Table 1:** Characteristics and genotype distributions in asthmatics and controlsª

	**Asthmatics**	**Controls**	**P-value**	**OR**	**95% CI**
Age (Mean ± SD), years	7.65 ± 1.916	8.27 ± 1.785	0.025*	_	_
Sex				_	_
Male	62 (66%)	44 (48.9%)	0.019*		
Female	32 (34%)	46 (51.1%)			
Spirometry			_	_	_
FEV1 (%predicted)**	97 ± 6.2	_			
FVC (%predicted)***	96 ± 8.2				
Disease severity					
Mild persistent	21 (22%)	_	_	_	_
Moderate persistent	36 (38.3%)				
Severe persistent	37 (39.3%)				
Atopy					
Atopic	67 (71.3%)		_	_	_
Non atopic	27 (28.7%)	90 (100%)			
Passive smoking exposure	39 (41.5%)	28 (31.1%)	0.168	_	_
GSTT1					
Null	82 (87.2%	88 (97.8%)			
Present	12 (12.8%)	2 (2.2%)	0.007*	0.683	0.034-0.715
GSTM1					
Null	47 (50%)	55 (61.1%)			
Present	47 (50%)	35 (38.9%)	0.130	1.000	0.539-1.857

ªData are expressed as no. (%) of patients unless otherwise indicated.

*P-value less than 0.05 is considered statistically significant.

**FEV1: forced expiratory volume in 1 second, ***FVC: forced vital capacity.

**Table 2:** Genotype distributions in atopic asthmatics and non-atopic asthmaticsª

	**Atopic asthmatics**	**Non-atopic asthmatics**	**P-value**
	**n = 67 (%)**	**n = 27 (%)**	
GSTT1			
Null	59 (88.1%)	23 (85.2%)	
Present	8 (11.9%)	4 (14.8%)	0.706
GSTM1			
Null	33 (49.3%)	14 (51.9%)	
Present	34 (50.7%)	13 (48.1%)	0.820

ªData are expressed as no. (%) of patients unless otherwise indicated.

**Table 3:** Genotype distributions in asthmatics stratified by disease severityª

	**Mild persistent**	**Moderate persistent**	**Severe persistent**	**P- value**
	**n = 24 (%)**	**n =36 (%)**	**N = 34 (%)**	
GSTT1				
Null	21 (87.5%)	29 (80.6%)	32 (94.1%)	
Present	3 (12.5%)	7 (19.4%)	2 (5.9%)	0.236
GSTM1				
Null	12 (50%)	19 (52.8%)	16 (47.1%)	
Present	12 (50%)	17 (47.2%)	18 (52.9%)	0.892

ªData are expressed as no. (%) of patients unless otherwise indicated.

## Discussion

It was recently recognized that GSTs play an active role in oxidative defenses and members of this superfamily may be determinants of respiratory health [10]. The presence of the GSTT1 null polymorphism was 87.2% and 97.2% in asthmatics and controls respectively and the results showed to be significantly higher in controls (P = 0.007, OR:0.683, CI: 0.034 -0.715). The results for GSTM1 were 50% and 61.1% respectively and showed to be nonsignificant (p = 0.130, OR: 1.000, CI: 0.54- 1.86). A systematic review and meta-analysis for the effects of GST genes on asthma demonstrated that a large Avon Longitudinal Study of Parents and Children found a protective effect on asthma of the GSTT1 null allele in mothers (OR: 0.71; 95% CI: 0.57–0.90 and 0.84; 0.63–1.12, for heterozygotes and null homozygotes, respectively, compared with wild-type homozygotes) and children (0.91; 0.76–1.11 and 0.89; 0.70–1.13) [11]. No association of the disease with GSTT1 null genotype was noted by several studies in different populations [2,12-20]. However, in contrary to our findings an increased risk was seen in individuals with this genotype in some studies [3,21-24]. Similarly, a study performed on Egyptian population in Zagazig -located in northern Egypt- showed that asthmatic children had a significant lower prevalence of GSTT1 null genotype than the control group (P = 0.003). However, a higher prevalence of the GSTM1 null genotype was observed in the asthmatic group [25]. As our study was performed in Cairo- the capital of Egypt located in the centre- the enrolled patients were referred from upper Egypt that’s why a difference in GSTM1 null genotype frequencies among asthmatics and controls in the two studies.

In agreement with the current study results, no association of the disease with GSTM1 null genotype was noted by several studies in different populations [2,12-16,18]. However, an increased risk was seen in individuals with this genotype in some reports [3,17,19-24]. The fact that asthma pathogenesis is a result of interactions between multiple genetic and environmental factors highlight that exposure to environmental chemical agents may explain the differences encountered.

A recent study done in Italy for asthmatic adults- which revealed no association for both null genotypes with the disease- stated that the differences in the prevalence of asthma in different ethnic groups reflect and highlight genetic variances with a significant coverage of environmental conditions. The study declared that rapid change in asthma prevalence is not linked to genetic changes in populations because these mechanisms are too slow to explain this scenario and the effect of environmental exposures and interactions between genetic factors and environmental conditions are still a matter of debate [26].

The current study revealed that the frequencies of GSTT1 and GSTM1 null genotypes in controls were 97.8% and 60.1% respectively. The frequencies previously reported in the Egyptian population (15% and 44% respectively among 34 subjects) [27] and in another study a higher frequencies was reported (25.50% and 55.50% respectively) [28]. According to our results, the frequency of the GSTM1 null genotype (60.1%) was slightly higher than the previous Egyptian studies, comparable to Caucasians (50.4% -58%), Europeans (39.00–62.00%) and White Americans (35.00–62.00%). However, the frequency of the GSTT1 null genotype (97.8%) was extremely higher than the range of the previous Egyptian studies, Europeans (10.00–21.00%), Africans (15.00–26.00%) and Caucasian- Americans (10.00–36.00%).

Differences in gene frequencies among various ethnic groups, may explain the differences encountered. A previous study that attempted to investigate the prevalence of important allelic variants of several genes including GST gene in the Egyptian population denoted that Egypt is unique geographically, as it is located centrally to the three continents of Africa, Europe and Asia, so its population is highly affected by the rapid pace of intercontinental transportation and large-scale immigration and that throughout history, the Greeks, Romans, Arabs, Turks, French and British have all ruled Egypt and mixed with its people, so that modern Egypt now is an amalgam of all these legacies. So, there is a considerable genetic admixture in the Egyptian population [28]. This genetic admixture explains more the differences encountered between the results of the current study done in Cairo- central Egypt- and that performed in Zagazig- northern Egypt.

The present study compared the GSTT1and GSTM1 null genotypes among asthmatic patients with the levels of asthma severity whether mild (87.5% and 50% respectively), moderate (80.6% and 52.8% respectively) or severe persistent (94.1% and 47.1% respectively) and it revealed no statistical significance (P value: 0.236 and 0.892 respectively). These findings were supported by several studies [12,14,15,29,30].

Regarding the genetic polymorphisms of GSTT1 and GSTM1 among atopic asthmatic patients and non-atopic asthmatics, no significant statistical difference in GSTT1 and GSTM1 polymorphisms was noted between the two groups (P value: 0.706 and 0.820 respectively). In agreement with the current study, a study done on Egyptian population in zagazig announced that there is no significant association found between atopy and GSTT1 polymorphism. However, they found that the GSTM1 null genotype was significantly higher in atopic asthmatic cases than in nonatopic asthmatic subjects (P = 0.01) [25].

Comparison of our results with other studies indicates that GSTT1 and GSTM1 null genotypes were not universally associated with the asthma phenotypes. The genetic basis of asthma may differ between different ethnic groups. Future studies of large size should focus on interactions of GST genes with environmental oxidative exposures and with other genes involved in antioxidant pathways. Quality of study conduct and reporting need to be improved to increase credibility of the evidence accumulating over time.

## Competing interests

The authors declare that they have no competing interests.

## Authors’ contributions

NMR, NMD and MMW - Designed, conducted and analyzed the study, NAM and NMR - Analyzed the data and drafted the manuscript. All authors revised and approved the manuscript.
